# Evidence That Aberrant Expression of Tissue Transglutaminase Promotes Stem Cell Characteristics in Mammary Epithelial Cells

**DOI:** 10.1371/journal.pone.0020701

**Published:** 2011-06-08

**Authors:** Anupam Kumar, Hui Gao, Jia Xu, James Reuben, Dihua Yu, Kapil Mehta

**Affiliations:** 1 Department of Experimental Therapeutics, The University of Texas MD Anderson Cancer Center, Houston, Texas, United States of America; 2 Department of Hematopathology, The University of Texas MD Anderson Cancer Center, Houston, Texas, United States of America; 3 Department of Molecular and Cellular Oncology, The University of Texas MD Anderson Cancer Center, Houston, Texas, United States of America; 4 Graduate School of Biomedical Sciences, The University of Texas Health Science Center, Houston, Texas, United States of America; University of Nebraska Medical Center, United States of America

## Abstract

Cancer stem cells (CSCs) or tumor initiating cells (TICs) make up only a small fraction of total tumor cell population, but recent evidence suggests that they are responsible for tumor initiation and the maintenance of tumor growth. Whether CSCs/TICs originate from normal stem cells or result from the dedifferentiation of terminally differentiated cells remains unknown. Here we provide evidence that sustained expression of the proinflammatory protein tissue transglutaminase (TG2) confers stem cell like properties in non-transformed and transformed mammary epithelial cells. Sustained expression of TG2 was associated with increase in CD44^high^/CD24^low/-^ subpopulation, increased ability of cells to form mammospheres, and acquisition of self-renewal ability. Mammospheres derived from TG2-transfected mammary epithelial cells (MCF10A) differentiated into complex secondary structures when grown in Matrigel cultures. Cells in these secondary structures differentiated into Muc1-positive (luminal marker) and integrin α6-positive (basal marker) cells in response to prolactin treatment. Highly aggressive MDA-231 and drug-resistant MCF-7/RT breast cancer cells, which express high basal levels of TG2, shared many traits with TG2-transfected MCF10A stem cells but unlike MCF10A-derived stem cells they failed to form the secondary structures and to differentiate into Muc1-positive luminal cells when grown in Matrigel culture. Downregulation of TG2 attenuated stem cell properties in both non-transformed and transformed mammary epithelial cells. Taken together, these results suggested a new function for TG2 and revealed a novel mechanism responsible for promoting the stem cell characteristics in adult mammary epithelial cells.

## Introduction

Metastatic breast cancer is an aggressive, chemoresistant and essentially incurable disease with median survival duration of about 2 years after metastasis has been detected [1]. Understanding the molecular mechanisms involved in cancer metastasis is critical for developing new strategies for improving patient outcomes. Recent advances in understanding the biology of tumor progression and metastasis suggest that cancer stem cells (CSC) or tumor-initiating cells (TIC) are important players in the progression of primary tumors to metastatic disease [2]. Published studies suggest that breast CSCs/TICs are relatively resistant to both chemotherapy and radiation therapy [3]. Moreover, due to their self-renewal and differentiation abilities CSCs/TICs can regenerate various subpopulations of the original tumor and endow tumors with the ability to survive and sustain growth [4]. While various studies have confirmed the existence of CSCs/TICs in humans and animal models with breast cancer [4], [5], it remains to be determined whether these cells originate from resident tissue stem cells or from acquisition of oncogenic lesions in which more differentiated epithelial cells have dedifferentiated into a primitive stem cell like state.

Our previous studies have demonstrated that breast tumors and tumor cell lines selected for resistance to drugs or isolated from metastatic sites exhibit increased expression of the pro-inflammatory protein tissue transglutaminase [6]–[9]. In our quest to determine the significance of increased TG2 expression in cancer cells, we found that stable expression of TG2 induces epithelial-to-mesenchymal transition (EMT) in mammary epithelial cells [10]. The TG2-induced EMT is associated with enrichment of the CD44^high^/CD24^low/-^ cell population, the antigenic phenotype considered to have CSC/TIC characteristics in non-transformed and transformed mammary epithelial cells [11].

Based on these observations and the observation that expression of EMT in mammary epithelial cells is associated with acquisition of CSC/TIC characteristics [12], we initiated studies to determine whether TG2-induced EMT could promote stem cell like properties in mammary epithelial cells. Here we present, for the first time, evidence that TG2 expression promotes phenotypic plasticity and shifts the dynamic equilibrium from non-stem to stem cells by increasing the number of cells with stem cell like properties. These results may suggest novel strategies for targeting CSC/TIC subpopulations in tumors for improved clinical outcomes.

## Results

### TG2 expression confers CD44^high^/CD24^low^ phenotype in mammary epithelial cells

**Figure 1:**
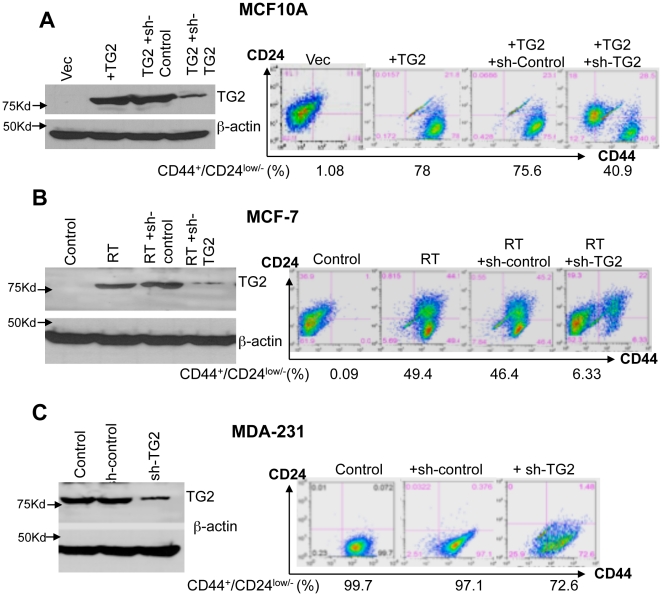
TG2 expression confers CD44^high^/CD24^low^ phenotype in non-transformed and transformed breast epithelial cells. (**A**) Immunoblot analysis (left panel) showing the expression of TG2 in vector- (MCF10A-Vec), TG2-lentiviral infected MCF10A cells (MCF10A-TG2) and MCF10A-TG2 cells infected with either control- (sh-control) or TG2-shRNA (shTG2). FACS analysis (Right panel) of cell-surface markers CD44 and CD24 in the cells described in left panel. (**B**) Immunoblot analysis (left panel) showing basal expression of TG2 in drug-sensitive (MCF-7) and drug-resistant (MCF7/RT) breast cancer cells and in MCF7/RT cells after their transfection with control or TG2-shRNA. FACS analysis (right panel) for cell-surface markers CD44 and CD24 in cells described in left panel. (**C**) Immunoblot analysis (left panel) showing basal expression of TG2 in highly aggressive MDA-231 breast cancer cells before and after their transfection with control- or TG2-shRNA. FACS analysis (right panel) for CD44 and CD24 antigen expression in cells described in left panel.

In our previous report we showed that stable expression of TG2 in mammary epithelial cells could induce the EMT and resulted in enrichment of CD44^high^/CD24^low^ subpopulation [10], the antigenic phenotype that is linked with normal and transformed mammary stem cells [11], [13]. To further address the significance of TG2 in promoting CD44^high^/CD24^low^ phenotype, we downregulated TG2 expression by small hairpin RNA (shRNA) and determined CD44/CD24 antigen expression. The MCF10A-TG2 cells with induced and MCF7/RT and MDA-231cells with constitutive TG2 expression were infected with TG2-shRNA or control-shRNA-lentiviral construct. Following the selection of stable clones against puromycin, cells were analyzed for CD44/CD24 expression. Results shown in Figure 1 demonstrated that downregulation of exogenous (MCF10A-TG2) or endogenous (MCF7-RT and MDA-231) TG2 is associated with a considerable decrease in the number of CD44^high^/CD24^low^ cells. No such change in control-shRNA transfected cells was evident. These observations further supported the contention that aberrant expression of TG2 in breast epithelial cells is associated with increased expression of stem cell antigenic markers.

### TG2 expression enhances mammosphere-forming ability in mammary epithelial cells

**Figure 2:**
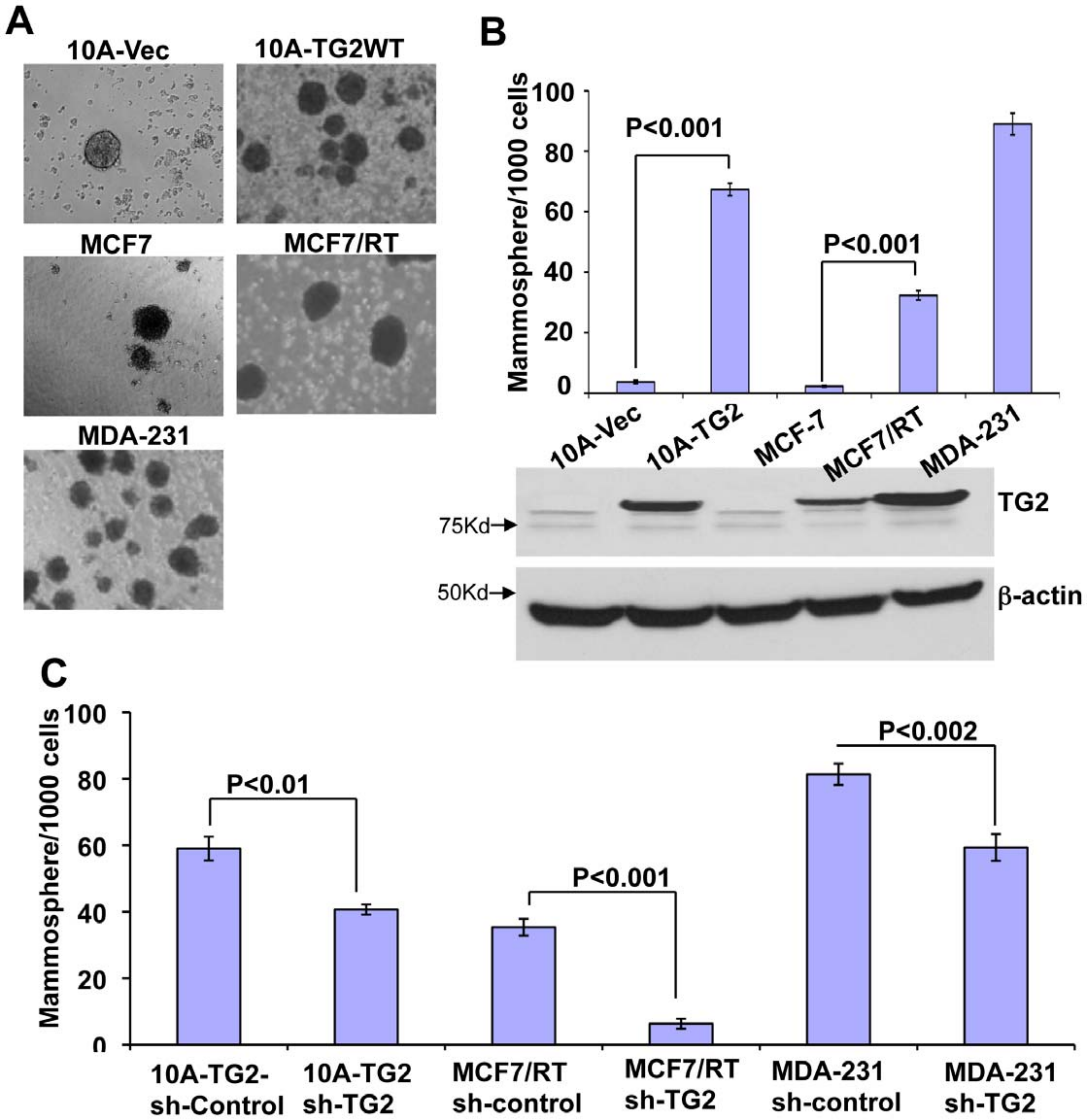
TG2 expression promotes mammosphere-forming ability in breast epithelial cells. (**A**) Phase-contrast images of mammospheres formed after 10 days' culture of indicated cells in the mammosphere medium. (**B**) Quantification of mammospheres formed by cells (top panel) and immunoblot analysis for TG2 expression (bottom panel) in cells described in (A). (**C**) Quantification of mammospheres formed by MCF10A-TG2, MCF7/RT and MDA-231 cells transfected with either control (sh-contol) or TG2 (sh-TG2) shRNA. The data shown are average number of mammospheres formed/1000 seeded cells ± SEM from triplicate values of a representative experiment performed three times with similar results.

Beside CD44^high^/CD24^low/-^ antigenic phenotype, the ability to withstand anoikis, to proliferate and differentiate in anchorage-independent manner, and to grow in clonal clusters or mammospheres are some additional characteristics of mammary epithelial stem cells [14], [15]. Implantation of *in vitro* generated mammospheres into the mammary stromal fat pad is able to reconstitute the entire mammary ductal tree [16], [17]. These observations imply that *in vitro* ability of cells to form mammospheres represents their self-renewal and gland-reconstituting abilities [15], [18], [19]. Therefore, we next examined the mammosphere forming ability of TG2 expressing mammary epithelial cells. Results shown in Fig. 2 demonstrated that neoexpression of TG2 in MCF10A cells was associated with >15-fold increase in mammospheres formation compared to the control vector-infected MCF10A cells (Fig. 2A and 2B). Similarly, the mammosphere forming ability of breast cancer cells with high basal TG2 expression (MCF7/RT and MDA-231) was significantly higher than the TG2-deficient cells (MCF-7) (Figure 2A and 2B). Importantly, downregulation of TG2 in these cells significantly compromised their ability to form memmospheres (Fig. 2C). These data further supported the significance of TG2 in conferring stem cell like attributes in mammary epithelial cells.

### TG2 expression augments the self-renewal ability

**Figure 3:**
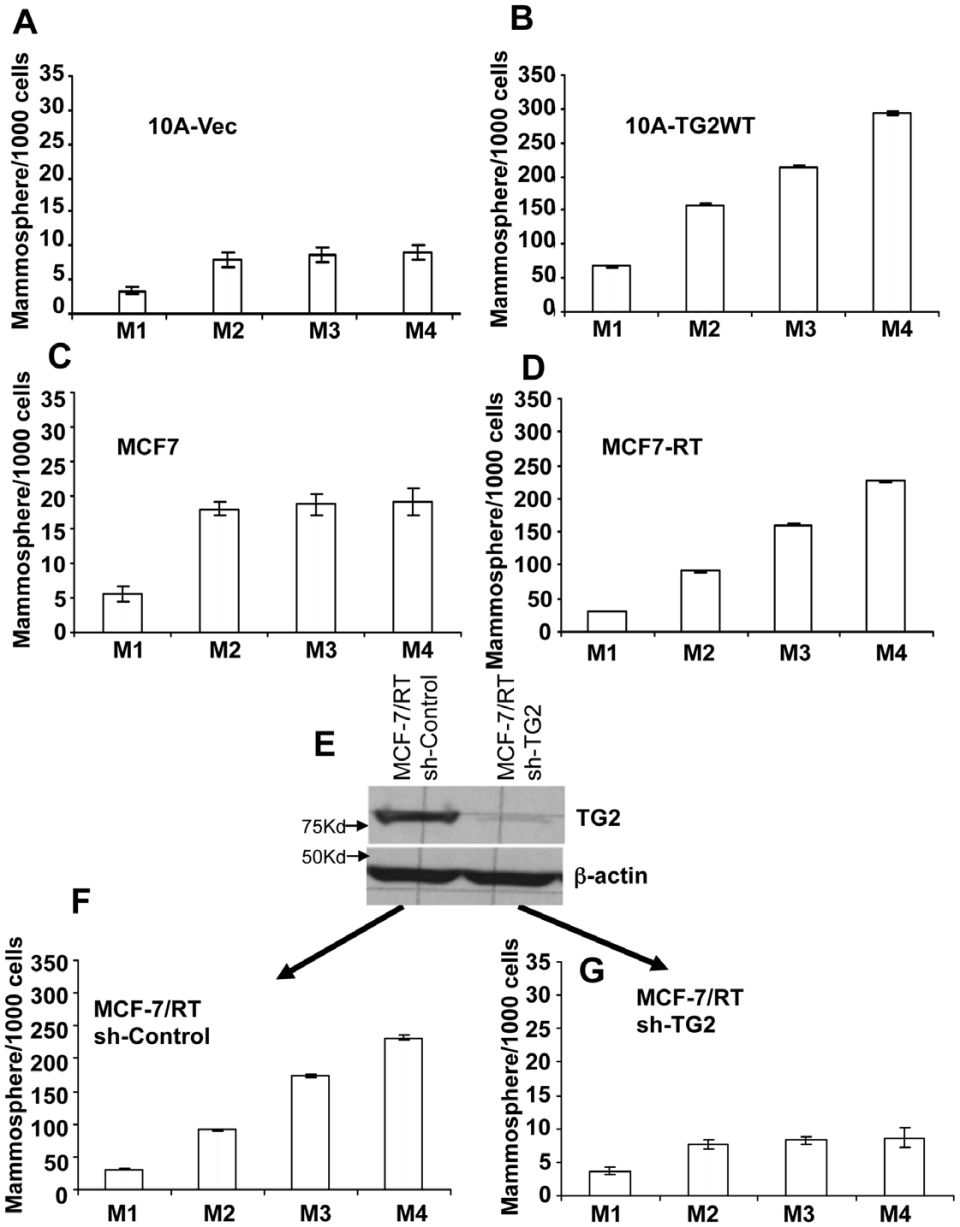
TG2 expression enhances the self-renewal ability of breast epithelial cells. To test the self-renewal ability, we assessed the sphere-initiation efficiency of serially passaged cells cultured as mammospheres for up to four generations. Number of mammospheres formed by (**A**) MCF10A-Vec, (**B**) MCF10A-TG2, (**C**) MCF-7 and (**D**) MCF-7/RT cells at different passages (M1 to M4). (**E**) Immunoblot analysis showing the expression of TG2 in MCF-7/RT cells transfected with either control or TG2 shRNA. Number of mammospheres formed by control- (**F**) and TG2-shRNA (**G**)-transfected MCF-7/RT cells during different passages (M1 to M4). The data shown are average number of mammospheres formed/1000 seeded cells ± SEM from triplicate values of experiments repeated twice with similar results. Please note 10-fold difference in y-axis scales for cells lacking TG2 (0–35) and TG2 expressing cells (0–350).

Stem cells have two distinct features – the ability of self-renewal and differentiation into multiple lineages. To test the self-renewal capability of TG2 expressing cells, we used an *in vitro* assay that relies on assessing the sphere-initiation efficiency of serially passaged cells cultured as mammospheres. Results shown in Figs. 3A and 3C revealed a 2-fold and 4-fold increase in the number of secondary mammospheres formed by culture of TG2-deficient MCF10A-Vec and MCF-7 cells, respectively. However, in subsequent passages the number of mammospheres formed remained steady (Fig. 3A and 3C) suggesting the maintenance of dynamic equilibrium between stem cells and non-stem cell populations. In contrast, the number of cells with mammosphere forming ability increased with each passage in TG2-induced MCF10A (Fig. 3B) and endogenously high TG2 expressing MCF7-RT (Fig. 3D) and MDA-231 cells (Fig. S1). These results suggested that constitutive expression of TG2 contributes to the increased ability of mammary epithelial cells for self-renewal. To further test this contention, TG2 expressing MCF7-RT cells were stably transfected with control or TG2-shRNA (Fig. 3E) and assayed for mammospheres forming efficiency in serially passaged cultures. Similar to the parental MCF-7/RT cells, control shRNA transfected (MCF-7/RT sh-Control) cells showed progressive increase in the number of mammosphere initiating cells with each passage (Fig. 3F). TG2-shRNA transfected cells (MCF7-RT sh-TG2) in contrast, showed ∼ 2-fold increase in secondary mammospheres and the proportion of mammosphere-forming cells remained unchanged in subsequent passages (Fig. 3G). Overall, these findings suggested that TG2 expression increased the cellular plasticity and shifted the dynamic equilibrium from non-stem cells towards stem cell compartment.

### TG2-induced stemness is a dynamic process

**Figure 4:**
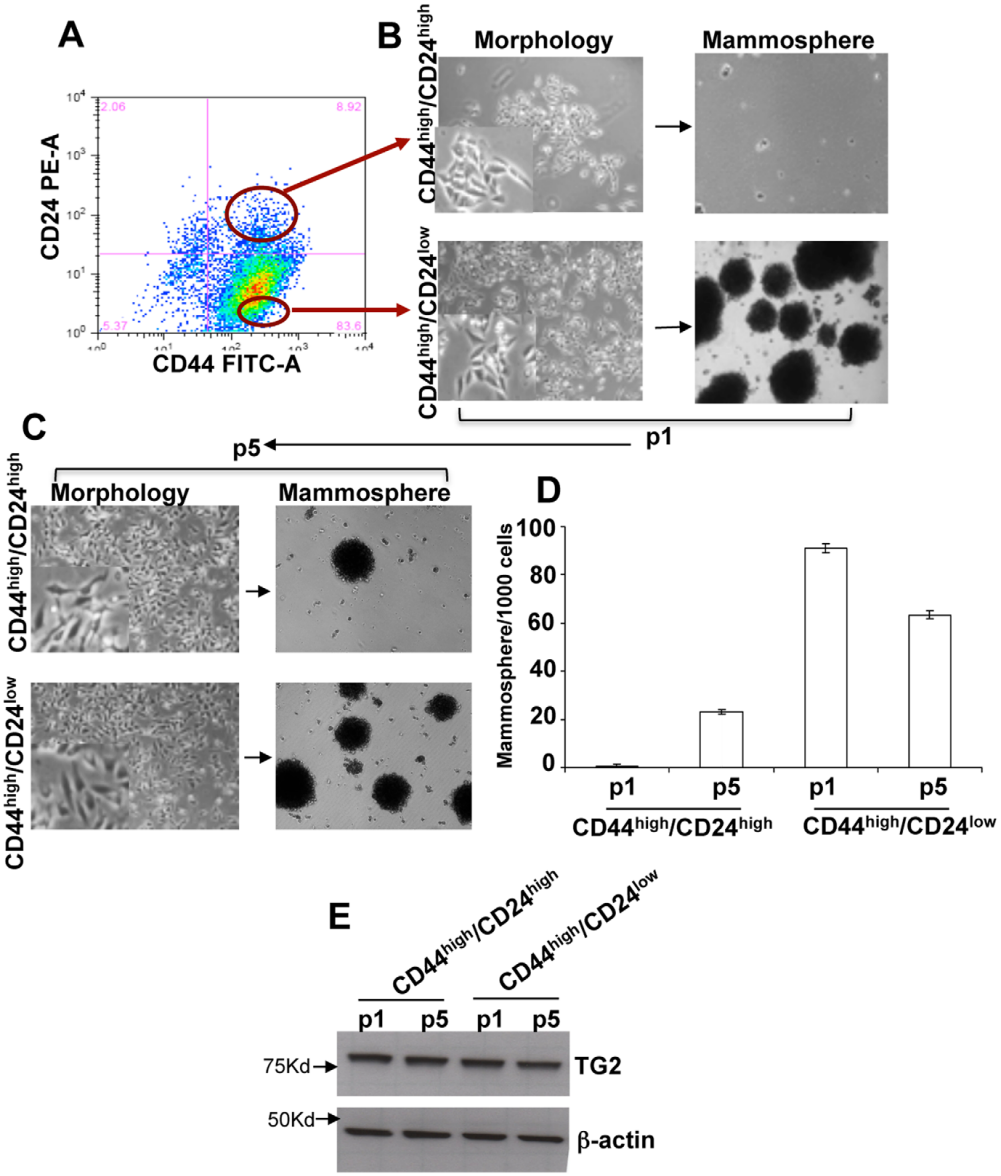
TG2 induced stem cellness is dynamic in nature. (**A**) CD44^high^/CD24^high^ cells and CD44^high^/CD24^low^ cells were separated by FACS. (**B**) Phase-contrast images of morphology (left) and mammospheres (right) obtained after seeding the CD44^high^/CD24^high^ (top) and CD44^high^/CD24^low^ (bottom) cells from (A). Magnification x100; inset, X500 (**C**) Phase-contrast images of morphology (left) and mammospheres (right) obtained by seeding CD44^high^/CD24^high^ (top) and CD44^high^/CD24^low^ (bottom) cells at the 5^th^ passage (p5). (**D**) Quantification of mammospheres formed by p1 and p5 cells from the sorted CD44^high^/CD24^high^ and CD44^high^/CD24^low^ populations. The data shown are average number of mammospheres formed/1000 seeded cells ±SEM from triplicate values of experiments repeated twice with similar results (**E**) Immunoblot analysis for TG2 expression in p1 and p5 cells from the sorted CD44^high^/CD24^high^ and CD44^high^/CD24^low^ populations.

It is generally believed that non-transformed mammary epithelial cells show lineage hierarchy in which CD44^high^/CD24^low^ cells (Stem cells) irreversibly differentiate into CD44^high^/CD24^high^ cells (non-Stem cells) [20]. Since TG2 expression resulted in cellular plasticity, next we determined weather its expression maintains such lineage hierarchy or not. The CD44^high^/CD24^low^ and CD44^high^/CD24^high^ subpopulations were purified from TG2 overexpressing MCF10A cells by FACS sorting (Fig. 4A) and purified subpopulations were allowed to grow in monolayer cultures. Morphologically the cells in two subpopulations looked identical with mesenchymal appearance (Fig. 4B). To evaluate their stem cell characteristics, we allowed these cells (p1) to grow in mammosphere cultures. Results shown in Fig. 4B demonstrated that CD44^high^/CD24^low^ cells were highly efficient in forming the mammospheres whereas CD44^high^/CD24^high^cells under identical conditions failed to grow into mammospheres (Fig. 4B and 4D). Moreover, the number of mammospheres formed by CD44^high^/CD24^low^ MCF10A-TG2 cells were higher than the unsorted MCF10A-TG2 cells (Fig. 2B). Next we allowed the sorted cells to grow in monolayer cultures for 15 days (p5). Once again, the cells in two subpopulations after 15 days of culture appeared similar with mesenchymal morphology (Fig. 4C). However, assay of these cells for CD44/CD24 expression revealed some rather surprising results. More than 80% cells in both the subpopulations showed CD44^high^/CD24^low^ phenotype (Fig. S2) and they were able to form the mammospheres (Fig. 4C). The number of mammosphere formed by CD44^high^/CD24^High^ cells increased while in CD44^high^/CD24^low^ cells it decreased after culture in monolayers (Fig. 4D). These results suggested that in presence of constitutive TG2 expression (Fig. 4E), CD44^high^/CD24^high^ (non-stem) cells have the ability to de-differentiate into stem cells and CD44^high^/CD24^low^ (stem cell) cells can differentiate into non-stem cells (Fig. 4E). Overall these observations support that constitutive expression TG2 increases the phenotypic plasticity and that TG2-induced stem cellness is dynamic in nature.

### TG2 expression does not affect the differentiation ability of mammary stem cells

**Figure 5:**
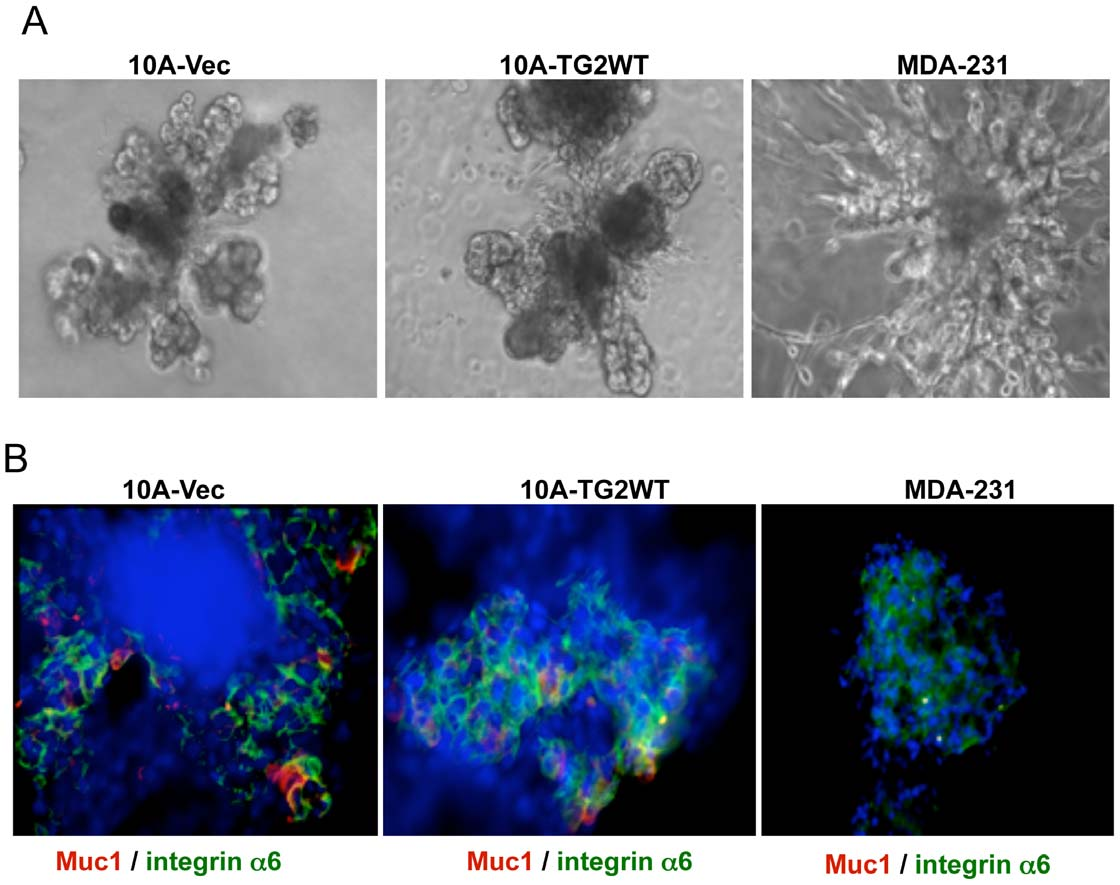
TG2 expression does not affect the differentiation ability of mammary stem cells. Mammospheres formed by indicated cell type were subsequently grown for 12 days in Matrigel containing prolactin (2 µg/ml). Differentiated structures were immunostained for basal (CD49f/integrin α6) and luminal (Muc1) cell markers. (**A**) Phase-contrast images of secondary structures formed by Matrigel cultures of indicated mammospheres in the presence of prolactin. (**B**). The differentiated structures from 10A-Vec and 10A-TG2-derived mammospheres show the presence of luminal (red), basal (green), and bipotent (yellow) cells. MDA-231 mammospheres, in contrast, under these conditions failed to differentiate into secondary structures (A) and did not show luminal marker expression but showed only low basal cell marker expression.

Another important characteristic of stem cells is their ability to differentiate into multiple lineages. To test the ability of TG2-expressing cells to differentiate and form secondary structures [14], we transferred individual mammospheres into Matrigel culture containing prolactin. Under these culture conditions the mammospheres derived from ‘empty vector’ or TG2-transfected MCF10A cells differentiated and formed complex secondary structures representing mammary gland like organotypic outgrowths (Fig. 5A). Immunostaining of these secondary structures revealed the presence of both Muc1 (luminal marker) and CD49f/integrin α6-positive (basal marker) cells (Fig. 5B) suggesting that TG2 expression doesn't effect the terminal differentiation of mammary stem cells in response to prolactin treatment. In contrast to MCF10A cells the mammosphere derived from breast cancer MDA-231 cells failed to differentiate into secondary structures, instead they grew into tumor-like mass with cells invading through the surrounding Matrigel (Fig. 5A). Moreover, immunostaning of MDA-231-derived secondary structures showed only faint staining for CD49f/integrin α6-positive (basal marker) cells and did not show any Muc1 (luminal marker)-positive cells (Fig 5B). These results suggested that TG2-induced stemness in non-transformed mammary epithelial cells maintains their ability to terminally differentiate whereas in cancer cells, TG2 expression though shifts the dynamic from non-stem to stem cells but these cells lack the commitment to terminally differentiate.

## Discussion

Consistent with our previous observations that increased expression of TG2 induces EMT in non-transformed and malignant mammary epithelial cells [10], here we found that TG2-induced EMT promotes stem cell traits in these cells. Thus, TG2 expression was associated with increased CD44^high^/CD24^low^ antigenic phenotype, increased ability to form mammospheres, increased self-renewal ability and differentiation into luminal and basal cell types. The first three features were shard by both the TG2-induced non-transformed MCF10A cells and transformed MCF7/RT and MDA231 cells, which express high basal levels of TG2. However, unlike MCF10-TG2 cells the ability of TG2 expressing MDA-231 cells to differentiate in response to prolactin treatment was lost.

These findings suggest a novel mechanism underlying the progression of tumor cells to aggressive and invasive phenotype. Thus, aberrant expression of TG2 in tumor cells promotes metastasis by inducing EMT and stemness, which in turn endorses the ability to cancer cells to migrate and grow at secondary sites. Indeed, it has been proposed that EMT can enable cancer cells not only to disseminate but also to acquire the ability of self-renewal by inducing stem cell state [20]. Interestingly, unlike non-transformed mammary epithelial cells, TG2 expressing cancer cells were unable to undergo differentiation in response to prolactin treatment. The lack of differentiation by transformed cells may reflect the function of some oncogenic mutation(s) or some other, yet unknown, defect in these cells. Creighton et al. [21] recently observed that the residual breast tumor cells in clinical samples surviving after conventional treatment are enriched in subpopulations of cells with both tumor-initiating and mesenchymal features, further supporting the link between EMT and stem cell phenotype. Our results also provide the evidence that differentiation of stem cells to non-stem cells and *vice versa* is a dynamic process. More recently, Illopoulos et al. [22] arrived a similar conclusion supporting that tumor heterogeneity involves a dynamic equilibrium between stem and non- stem cell compartments and this plasticity is regulated by interlukin (IL)-6, the inflammatory cytokine that is known to induce the expression of TG2 in various cell types [23], [24]. Based on these observations, it is tempting to speculate that IL-6-induced stemness in mammary epithelial cells may be related to its ability to upregulte TG2 expression. Indeed, in a related system we recently showed that the proinflammatory cytokine TGF-β could induce the EMT by virtue of its ability to upregulate TG2 expression [10].

These results suggest that phenotypic plasticity in tumor cells may result from dynamic equilibrium that exists between CSCs and non-CSCs [25], [26]. Moreover, this equilibrium may shift in one or another direction in context to specific signals within the tumor microenvironment that influence the probability of interconversion of CSC and non-CSC compartments [27]. Moreover, our data provide additional evidence supporting that constitutive expression of TG2 contributes to the increased phenotypic plasticity in mammary epithelial cells.

Many primary epithelial tumor cell types rely on the EMT program to facilitate the execution of successful invasion-metastasis cascade [28]. Earlier we and other groups have observed that aberrant expression of TG2 is associated with metastatic and drug resistance phenotypes in breast cancer cells [6]–[9], [29], [30]. In line with these observations, we recently found that TG2 expression induces EMT in non-transformed and malignant mammary epithelial cells and facilitates the execution of most steps in the invasion-metastasis cascade [10]. However, the last step, termed colonization, which involves the growth of micrometastases into macroscopic metastases, has represented a conceptual dilemma; if vast majority of cells leaving the primary tumor and disseminating to distant sites lack self-renewal capability, their ability to form macroscopic metastases can be compromised because of limited proliferative potential. Our current findings address this dilemma and suggest that TG2-induced EMT may not only enable cancer cell to disseminate from the primary tumors but also endow many of the properties of stem cells such as, expression of CD44^high^/CD24^low^ antigenic phenotype, ability to form mammosphere, self -renew ability and multilineage differentiation. Importantly, we found that aberrant expression of TG2 enhances the self-renewal ability in both normal and malignant mammary stem cells and shifts the dynamic equilibrium from non-stem cells towards stem cells. In contrast, downregulation of TG2 not only compromised the self-renewal ability of cells but also restored the homeostasis of dynamic equilibrium between stem and non-stem cells. The observation that self-renewal ability of cancer cells is critical for the successful formation of macroscopic metastasis in conjunction with the current observation that TG2 promotes the self-renewal ability, further supported our earlier observations that metastatic breast tumors express high basal levels of TG2 [6].

To our knowledge, these data provide the first evidence supporting that TG2-expression in mammary epithelial cells bestows stem cell traits (CD44^high^/CD24^low/-^ phenotype, ability to form mammospeheres, self-renewal, and differentiation into basal and luminal cells). Similarly, high basal expression of TG2 in breast cancer cells (MDA-231, MCF7/RT) though promotes certain stem cell properties (CD44^high^/CD24^low/-^ phenotype, ability to form mammospheres, and self-renewal ability) but failed to support their terminal differentiation. These results reveal a novel function of TG2 in cancer cells and suggest that its aberrant expression promotes the EMT and stem cell-like properties in mammary epithelial cells. Furthermore, these results also support earlier observations that TG2 expression is associated with the drug resistance and metastasis of breast cancer [6]–[8], hence TG2 represents a promising therapeutic target for the treatment of metastatic breast disease.

## Materials and Methods

### Cell lines and vectors

The immortalized human mammary epithelial cell line MCF10A and breast cancer cell lines MCF7, MCF7-RT and MDA-231 were maintained as previously described [7], [8], [10]. The open reading frame of *TG2* was subcloned into a pCDH lentiviral vector (System Biosciences, Frederick, MD) from a pcDNA3.1-TG2 vector (kindly provided by Dr. Gail Johnson, University of Rochester, NY). 10A-Vec and 10A-TG2 cells were established by retroviral infection of MCF10A cells as described previously [10]. Stable clones were selected against 800 ng/ml puromycin. Multiple stable clones were used to rule out potential clonal effects. All experiments were performed between passages 5 and 25. MDA-231, MCF7/RT and 10A-TG2 cells were transfected with control-shRNA or TG2-shRNA lentiviral particles according to instructions provided by the supplier (Santa Cruz, CA).

### FACS analysis

The anti-CD44 (clone G44-26) and anti-CD24 (clone ML5) antibodies used for fluorescence-activated cell sorting (FACS) analysis were obtained from BD Biosciences (San Jose, CA). Briefly, for each cell line, 5×10^5^ cells were aliquoted into 2 tubes; tube 1 was stained with IgG isotype controls for FITC and PE; tube 2 was stained with anti-CD44-FITC and anti-CD24-PE. Cells were incubated with the appropriate antibodies at room temperature in the dark for 30 minutes and then washed with PBS. Cells were analyzed using a 4-color FACSCalibur flow cytometer (BD Biosciences); each sample recquired 10,000 cells for analysis. To separate CD44^high^/CD24^low^ (stem cells) from CD44^high^/CD24^high^ (non stem cells) MCF10A cells, flow cytometric cell sorting was performed on single-cell suspensions that were stained with CD44 and CD24 antibody.

### Mammosphere culture

Mammosphere were cultured as described in Dontu et al. [14], except that the culture medium (serum-free MammoCult® medium from Stem Cell Technology, Vancouver, Canada) contained 0.9% methylcellulose to prevent cell aggregation. The mammospheres were cultured for 7–10 days and mammospheres with diameter >75 µm were counted. For serial passages, mammospheres were collected by gentle centrifugation (800 rpm) and dissociated enzymatically (10 min in 0.05% trypsin, 0.53 mM EDTA) and mechanically, using a fire-polished Pasteur pipette. The dissociated cells were passed through a 40-µm sieve and checked under a light microscope for single-cellularity. One thousand dissociated cells were plated in a 6-well ultra-low attachment plate and cultured for 10 days. To check the self-renewal ability of the cells, mammospheres derived from two-dimensional culture were serially passaged for up to four generations in the mammosphere medium.

### Mammosphere differentiation and immunofluorescence

The mammosphere differentiation assay was adopted from Dontu et al. [14] and Weaver and Bissell [31]. Essentially, mammospheres were collected by gentle centrifugation (800 rpm); washed twice with PBS to remove the methylcellulose; resuspended in mammary epithelial cell growth medium (Clonetics, San Diego, CA) supplemented with 4% Matrigel (BD Biosciences), 10 ng/ml fibroblast growth factor (BD Biosciences), 4 ng/ml heparin (Sigma, St Louis, MO), and 2 µg/ml prolactin (Invitrogen, Carlsbad, CA); plated on eight-chambered slides pre-coated with Matrigel and allowed to grow for an additional 10–12 days. The structures generated by Matrigel culture were then photographed.

For immunofluorescence staining of the cell structures, Matrigel was first dissolved by incubation with gel recovery solution (BD Biosciences) for 2 hr at 4°C. Subsequently, the dissolved Matrigel and remaining media were gently removed, and the structures were fixed with 2% buffered formalin in PBS for 20 minutes at room temperature and washed three times with PBS containing 100 mM glycine. Cells were permeabilized with 0.5% Triton X-100 and blocked with 10% goat serum in immunofluorescence wash buffer for 1 hr at room temperature and incubated with primary anti-CD49f/integrin α6 (GoH3, Pharmingen, San Diego, CA) or anti-Muc1 (HMPV, Pharmingen) antibody overnight at 4°C. Subsequently, samples were washed three times with immunofluorescence wash buffer and incubated with Alexa-conjugated secondary antibodies (Invitrogen) for 1 hr at room temperature, and washed three more times with wash buffer. For nuclear staining, cells were incubated with 5 ng/ml 4′-6-Diamidino-2-phenylindole (DAPI) in PBS for 10 min, washed with PBS, and mounted with Prolong antifade mount (Invitrogen). Slides were viewed under a Laser-Scanning Confocal microscope (Olympus, Hicksville, NY).

### Western blotting

Cells were lysed in 50 mM Tris-HCl buffered saline (pH 7.5), containing 0.5% NP-40 on ice. Fifty micrograms of total protein from each sample was resolved on a 4%–12% bis-Tris gel with MOPS Running Buffer (Invitrogen) and transferred to Polyvinylidene fluoride membranes (PVDF). The membranes were then probed with appropriate primary and secondary antibodies.

## Supporting Information

Figure S1 **Number of mammospheres formed by MDA-231 cells at different passages (M1 to M4).** The data shown are average number of mammospheres formed/1000 seeded cells ± SEM from triplicate values from a representative experiment, repeated twice with similar results.
(TIF)

Figure S2 **FACS analysis of cell-surface markers CD44 and CD24 during passage p5 of cells sorted for CD44^high^/CD24^high^ and CD44^high^/CD24^low^ subpopulations.**
(TIF)
