# Supplementary figures and images for: Evidence That Aberrant Expression of Tissue Transglutaminase Promotes Stem Cell Characteristics in Mammary Epithelial Cells
Source: PLoS One. 2011 Jun 8;6(6):e20701. doi: 10.1371/journal.pone.0020701 (PMC3110765; doi:10.1371/journal.pone.0020701)

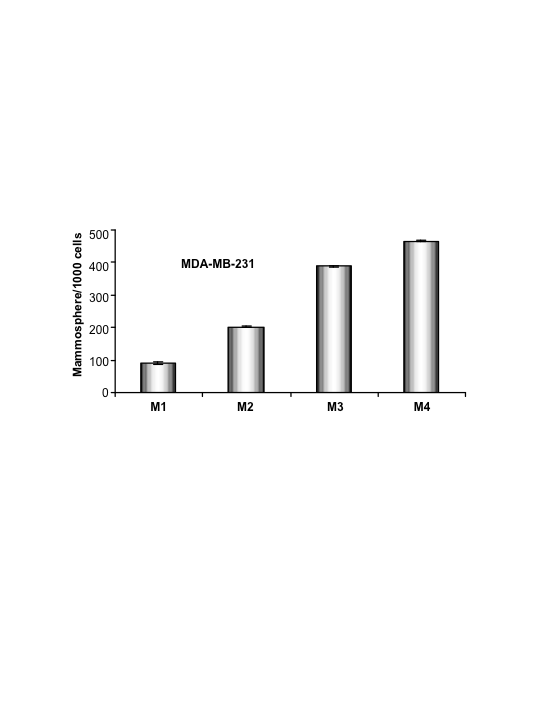

Supplement: Figure S1 — Number of mammospheres formed by MDA-231 cells at different passages (M1 to M4). The data shown are average number of mammospheres formed/1000 seeded cells ± SEM from triplicate values from a representative experiment, repeated twice with similar results. (TIF) [file pone.0020701.s001.tif]

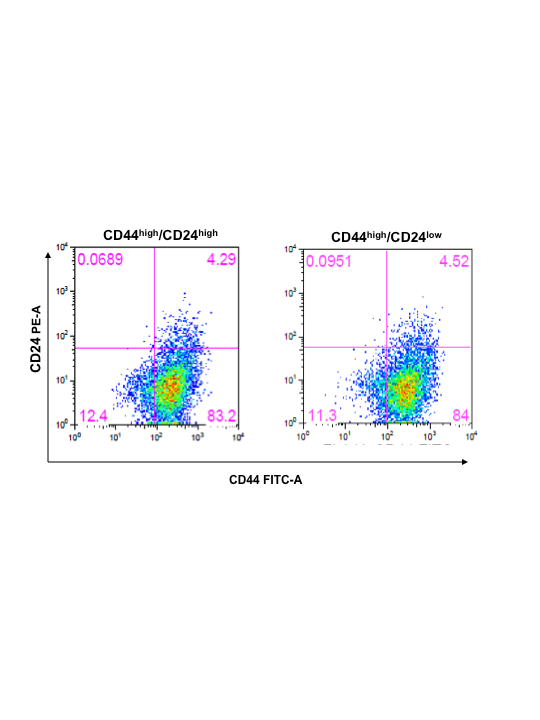

Supplement: Figure S2 — FACS analysis of cell-surface markers CD44 and CD24 during passage p5 of cells sorted for CD44high/CD24high and CD44high/CD24low subpopulations. (TIF) [file pone.0020701.s002.tif]
